# Implementing a regional School Health Research Network in England to improve adolescent health and well-being, a qualitative process evaluation

**DOI:** 10.1186/s12889-023-15713-9

**Published:** 2023-04-23

**Authors:** Emily Widnall, Lorna Hatch, Patricia N Albers, Georgina Hopkins, Judi Kidger, Frank de Vocht, Eileen Kaner, Esther MF van Sluijs, Hannah Fairbrother, Russell Jago, Rona Campbell

**Affiliations:** 1 Population Health Sciences, Bristol Medical School, University of Bristol, Canynge Hall, Bristol, BS8 2PL England; 2 Faculty of Medical Sciences, Newcastle University, Newcastle upon Tyne, England; 3 MRC Epidemiology Unit, University of Cambridge, Cambridge, England; 4 Health Sciences School, University of Sheffield, Sheffield, England; 5 Centre for Exercise Nutrition & Health Sciences, School for Policy Studies, University of Bristol, Bristol, England

**Keywords:** Mental health, Well-being, Adolescents, Schools, School Health Research Network

## Abstract

**Background:**

There is an increased need for prevention and early intervention surrounding young people’s health and well-being. Schools offer a pivotal setting for this with evidence suggesting that focusing on health within schools improves educational attainment. One promising approach is the creation of School Health Research Networks which exist in Wales and Scotland, but are yet to be developed and evaluated in England.

**Methods:**

This qualitative process evaluation aimed to identify the main barriers and facilitators to implementing a pilot School Health Research Network in the South West of England (SW-SHRN). Semi-structured interviews were conducted with school staff, local authority members, and other key stakeholders. Interview data were analysed using the 7-stage framework analysis approach.

**Results:**

Four main themes were identified from the data: (1) ‘Key barriers to SW-SHRN’ (competing priorities of academic attainment and well-being, schools feeling overwhelmed with surveys and lack of school time and resource); (2) ‘Key facilitators to SW-SHRN: providing evidence-based support to schools’ (improved knowledge to facilitate change, feedback reports and benchmarking and data to inform interventions); (3) ‘Effective dissemination of findings’ (interpretation and implementation, embedding findings with existing evidence and policy, preferences for an online platform as well personalised communication and the importance of involving young people and families); and (4) ‘Longer-term facilitators: ensuring sustainability’ (keeping schools engaged, the use of repeat surveys to evaluate impact, informing school inspection frameworks and expanding reach of the network).

**Conclusion:**

This study identifies several barriers to be addressed and facilitators to be enhanced in order to achieve successful implementation of School Health Research Networks in England which include providing a unique offering to schools that is not too burdensome, supporting schools to take meaningful action with their data and to work closely with existing organisations, services and providers to become meaningfully embedded in the system.

**Supplementary Information:**

The online version contains supplementary material available at 10.1186/s12889-023-15713-9.

## Background

Adolescence offers a key opportunity for early intervention with preventive approaches to promote health and well-being across the life course [1, 2]. There is a clear and well-evidenced link between young people’s physical health, emotional health and well-being, and their cognitive development and learning [3–5]. Schools offer a pivotal setting for this with evidence suggesting that focusing on health within schools improves educational attainment [4–7].

International guidance has focused on adopting a whole school approach to young people’s health and well-being for several years, namely the World Health Organization’s (WHO) Health Promoting Schools (HPS) Framework [8] which has been re-advocated in recent years with WHO calling for making every school a health promoting setting [9]. Whole school approaches involve all parts of the school working together and sharing a commitment, ethos and culture towards health and well-being. The HPS Framework comprises of health education being addressed in the school curriculum, health and well-being promotion through changes to the school environment and schools engaging with families and communities to help strengthen these health messages. Public Health England published guidance on the 8 principles to promoting a whole school approach to mental health and well-being more specifically, which include; enabling student voice to influence decisions, working with parents and carers and identifying need and monitoring impact of interventions [3]. Literature on embedding whole-school approaches to health and well-being discusses developing supportive policy (e.g. anti-bullying), the potential for schools to re-shape their identity through prioritising values such as care, respect and empathy, as well as schools creating a culture that enables young people to feel confident talking about how they feel [10, 11]. Review-level evidence suggests that a whole-school approach is effective in encouraging healthy behaviours in young people including physical activity, healthy eating, and in prevention of tobacco use and bullying [12].

Despite growing recognition of school-based health improvement, there remain a number of barriers to improving health and well-being in this context, including financial constraints, schools focussing on educational outcomes and school performance and limited understanding about effective health interventions [13]. One established method for overcoming these barriers has been the creation of School Health Research Networks (SHRNs). SHRNs use a whole system approach to facilitate health improvement in schools in that it brings together stakeholders and communities to develop a shared understanding of how best to improve school-aged children’s health and well-being [14], a collaborative model that goes beyond typically commissioned school surveys. System-based approaches look at the interrelationships between components of a system (e.g. a school) and the broader system as a whole (e.g. wider educational and government systems) [15]. Although established SHRNs exist with the UK (SHRN, Wales; https://www.shrn.org.uk/ and SHINE Scotland; https://shine.sphsu.gla.ac.uk/) as well as internationally (COMPASS, Canada; https://uwaterloo.ca/compass-system/), a SHRN has yet to be implemented in England. These networks help schools work with researchers to generate and use good quality evidence regarding health improvement [16].

Each country has their own unique context and while we can learn from experiences of SHRNs in other countries, we cannot simply replicate what these networks have done and expect it to work in the same way. We therefore require country-specific research to understand the unique barriers and facilitators to developing and sustaining SHRNs. In comparison to Wales and Scotland, England has a diverse school system with a variety of school types including Grammar schools that select students based on academic achievement, Academy schools that are state-funded but independent from local authorities and therefore decide on their own curriculums, and Free schools which are similar to academies but run by charities. Only a very small proportion of schools in England are still maintained by local government (11%).

Academy schools, have autonomy over their national curriculum as well as how they support and teach about mental health and well-being [17].A recent qualitative study revealed a wide amount of variability amongst academy trust leaders in how they perceive the role of academies in promoting health and well-being amongst students [13]. This study also revealed differences in whether multi-academy trusts (those responsible for more than one school) adopt a centralised strategy to health promotion, or allow individual schools autonomy. Existing structures in England means that there are different decision making approaches for health and well-being in different schools and therefore a SHRN needs to be sufficiently flexible to fit in with these varying structures, and this research will help us understand how best to do this.

One existing study in England testing a similar model to a SHRN is the BeeWell study (https://gmbeewell.org/), an annual well-being survey of secondary school pupils across Greater Manchester. Although BeeWell have adopted a regional approach in England, Greater Manchester is a city-region with a combined authority (a group of two or more local government councils that collaborate/take collective action). SW-SHRN is more ambitious in that it is seeking to create a network across a larger geographic area, made up of 15 separate local government administrative areas. Therefore, we want to understand the barriers and enablers to doing this at scale.

Our pilot study created a network of 18 schools from 6 local authorities in the South West of England. This paper reports on a qualitative process evaluation of implementing this pilot network to determine the barriers and facilitators to inform the expansion and continuation of the existing pilot network. A working logic model of SW-SHRN can be found within the study protocol paper [18].

We aimed to answer the following four research questions:

i. What are the key issues that impact the successful delivery and running of the SW-SHRN?
ii. What key information is required by schools to maximise the impact of the SW-SHRN?
iii. What data does the SW-SHRN need to provide to be successful and informative?
iv. What is required for the SW-SHRN to be sustainable long term? (sustaining school recruitment, retention and sustaining partnerships to best support schools to improve student health and well-being)

## Methods

### Design and participants

This process evaluation forms part of a larger pilot study of the SW-SHRN in which Year 8 (age 12–13) and Year 10 (age 14–15) secondary school students (n = 5,211) participated in an online health and well-being survey in school time (within one school lesson)[18]. The survey topics included mental health and well-being, physical activity and eating behaviour, sexual health, risky behaviours (smoking and alcohol use), body image, sleep, peer support, cyberbullying, social media use and the school connectedness. Parental opt-out informed consent is obtained prior to the student survey as well as students providing informed consent at the beginning of the survey. Full methodological details can be found in the pilot study protocol paper [18]. Schools (n = 18) and local authorities (n = 6) receive tailored feedback reports on the student data and researchers worked closely with schools in order to suggest key areas in which to make changes and to facilitate sharing of best practice between schools across the South West of England.

This process evaluation was based on a series of semi-structured interviews with school staff, local authority members, and wider key stakeholders. The key school contact at each participating school (n = 18) was invited to participate in a feedback interview. This was the member of staff involved in organising and delivering the SW-SHRN student survey in school and involved in receiving feedback reports and working with the team to make changes. Local authority staff from participating and non-participating schools in South West England were also invited to participate in an interview, these staff had school specific roles and some staff supported recruiting schools to the network. Other key organisations and individual stakeholders were identified by the research team at the study outset; these consisted of staff within charities, government departments, universities, academy trusts, and local councils whose remit was to work with schools. Local authorities and wider stakeholders were approached by a member of the research team via email with an information sheet and consent form and invited to participate in an interview. For stakeholders who had no prior knowledge of the network, an overview of SW-SHRN was provided in advance of the interview. Participants all received a full information sheet and consent form to sign in advance of the interview taking place, where written consent was not received before the interview took place, verbal consent was taken (and recorded) before the interview began. Ethical approval for the study was granted by University of Bristol’s Faculty of Health Sciences Research Ethics Committee (Ref. 110,922).

### Data collection and analysis

EW, a female mixed methods public health researcher with experience in conducting qualitative interviews and mental health research in schools conducted the research interviews. All interviews took place either over the phone or via an online video conferencing platform (e.g. Microsoft Teams). Interviews followed a topic guide (additional file 1 & 2). The local authority and stakeholder topic guide included questions on stakeholder views on the network, their perceived barriers and facilitators, what outputs they would like to see from the network and how to make the network sustainable and scalable. The school staff topic guide included questions on school recruitment methods, experiences of participation, feedback on administering the student survey, views on tailored school reports, how they would use the data provided with their school and what would encourage them to continue being part of the network.

Interview data were analysed by EW and LH using Gale and colleagues 7-stage framework analysis approach [19]. NVivo version 12 software (QSR International) was used to aid data management [20]. Audio recordings were transcribed verbatim, reviewed, and checked for accuracy by EW prior to analysis (stage 1). All transcripts were initially read by EW to gain familiarity with the interview data, EW recorded any initial contextual notes or early interpretative thoughts. (stage 2). EW and LH then independently read and annotated six randomly selected transcripts; two school contact interviews, two local authority member interviews and two wider key stakeholder interviews to generate an initial list of codes and create a draft framework (stage 3). EW and LH then met to discuss and compare these initial codes and agree on a final set of codes to apply to the remaining interview transcripts. A draft analytical framework was then produced(stage 4). Although there were some distinct differences between school contact interviews compared to wider stakeholders, there was sufficient overlap to allow all transcripts to be coded using the same analytical framework. Our analytic framework was then applied to all remaining transcripts which were single-coded by either EW or LH(stage 5), with further regular discussions to expand or refine the framework as needed. Charting then took place which Gale and colleagues describe as ‘summarizing the data by category’ (p.5)[19]. EW and LH charted the data into the framework matrix by creating summaries and identifying key quotes to represent each category (stage 6). EW and LH met regularly to interpret the data, identifying central characteristics and comparing data categories between and within cases to generate a set of themes and subthemes (stage 7). The final set of themes and subthemes identified were then discussed, revised, and agreed by all co-authors.

Codes were both deductive (generated from our topic guide and research questions) and inductive (generated from interview data). The Framework Method was chosen due to its ability to incorporate both inductive and deductive codes as well as the strengths of the charting/matrix process embedded within this approach which ensured that researchers were able to pay close attention to describing the data of each organisation type (school, authority, government department etc.) before comparing similarities and differences across organisations. Charting also allows the views of each research participant to remain connected to other aspects of their account within the matrix which avoids losing the context of individual viewpoints [19].

The researchers conducting and analysing the interviews were working on a project that was focussed on the creation of a school health research network and it is therefore possible that were unconscious biases towards the promotion of the network in the interpretation of the data.

## Results

A total of 26 semi-structured interviews were conducted with key school contacts (n = 11 from 11 individual schools); local authorities (n = 5) and wider key stakeholders (n = 10). Table 1 summarises the interviews by organisation and role type.

**Table 1:** Summary of Key Stakeholder Interviews by Organisation and Role Type

Interview	Organisation Type	Role Type
KS1	Charity	Mental health lead
KS2	Government department	Mental health, national
KS3	Government department	Public health, national
KS4	Government department	Public health, regional
KS5	Government department	Research lead, national
KS6	Government department	Public health, national
KS7	University	Clinical Psychologist/Academic
KS8	Academy Trust	Governor
KS9	NHS	Mental Health Support Team
KS10	Government department	Mental health, regional
LA1	Local authority	Advanced Public Health Practitioner, Health & Well-being
LA2	Local authority	Health Improvement Specialist: Children & Young People
LA3	Local authority	Children & Families Commissioning
LA4	Local authority	Lead for Health and Well-being (Education and Learning) Services for Children and Young People
LA5	Local authority	Children and Families
SC1	Participating school	Deputy Head Teacher
SC2	Participating school	Pastoral Support Worker
SC3	Participating school	Deputy Head Teacher
SC4	Participating school	Head of Personal Development Curriculum
SC5	Participating school	Deputy Head Teacher, Student Welfare & Behaviour
SC6	Participating school	Music Teacher, Lead for Looked After Children
SC7	Participating school	Mental Health & Well-being Coordinator
SC8	Participating school	PSHE Lead
SC9	Participating school	Assistant Headteacher
SC10	Participating school	Deputy of PE and Health, PSHE Lead
SC11	Participating school	Deputy Head Teacher

*‘KS’ = key stakeholder, ‘LA’ = Local Authority ‘SC’ = School Contact, PSHE = Personal, social, health and economic education*

**Fig. 1:**
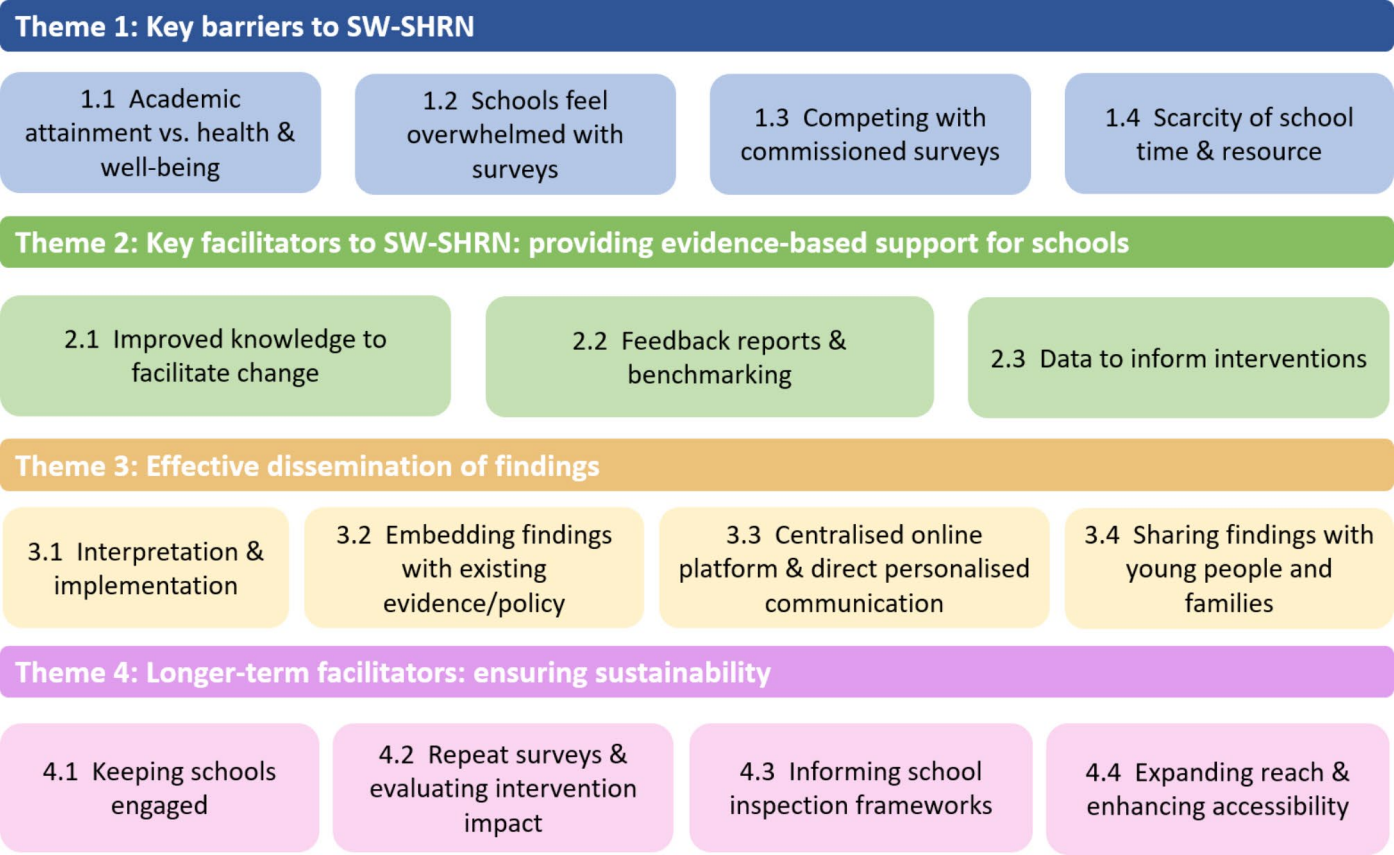
Barriers and facilitators to implementing a School Health Research Network in the South West of England

The four key themes identified from the data were (1) Key barriers to SW-SHRN; (2) Key facilitators to SW-SHRN: providing evidence-based support to schools; (3) Effective dissemination of findings; and (4) Longer-term facilitators: ensuring sustainability. Theme 1 and 2 relate to research question 1, identifying key issues that impact the successful delivery and running of SW-SHRN. Theme 3 relates to research questions 2 and 3 by identifying key information required by schools to maximise the impact of the network and identifying what data SW-SHRN needs to provide to be successful. Theme 4 relates to research question 4 and identifies what is required for SW-SHRN to be sustainable long-term. Figure 1 provides an illustrative overview of the four key themes and subthemes.

### Theme 1: key barriers to SW-SHRN

Stakeholders suggested a number of potential barriers to the successful roll out and growth of SW-SHRN which were divided into five subthemes: (1) academic attainment vs. health and well-being; (2) schools overwhelmed with surveys; (3) competing with commissioned surveys; (4) scarcity of school time and resource and (5) reduced role of local authorities. This theme discusses these five key barriers to SW-SHRN, as well as detailing suggested facilitators considered by stakeholders to reduce the impact of these barriers.

#### Academic attainment vs. health and well-being

Schools differed in their levels of priority for student health and well-being, with academic achievement and attendance remaining the central priority in schools. Stakeholders addressed the importance of continued communication about the strong links between health and attainment to schools.

*“Ultimately, schools get incentivised for academic achievements and for attendance. Therefore…when we are asking them to allow time and to prioritise other things…how do we argue the case for why this is beneficial? The evidence tells us that young people with depression have less school attendance, and do less well in terms of educational achievement…but how do we get schools to buy into that?” (KS 7)*.

Stakeholders also believed that communicating the link between health and attainment became particularly important when thinking about how to engage harder to reach schools.

*“I mean, if you are really getting into the harder to engage schools, then there might be more really strong links on why this is important for their academic outcomes, so being able to demonstrate that. That might be better than just why this is good for your kids’ health and well-being outcomes.” (KS 2)*.

#### Schools feel overwhelmed with surveys

Schools and stakeholders discussed the large volume of surveys currently being offered to schools, particularly because of the COVID-19 pandemic. Surveys discussed were not exclusively research surveys but also included surveys from councils, companies and charities. This has led to schools feeling overwhelmed with survey offers, and having to weigh up which ones to take part in and sometimes actively trying to reduce the number of surveys going out to students.

*“Schools get offered a lot of stuff. People will constantly, “Can we do this with you? There is this initiative.” Some of which are government backed, some of which are university backed, some of which come from elsewhere. They are… very, very busy and so miss stuff at times, even if it is good stuff, and even if they want to do it they are not able to do it.” (KS 8)*.

*“I think my view is, yes, there are a lot of surveys going on…the one area I’ve looked at is, can we reduce the amount of surveys that are going out?” (SC 10)*.

#### Competing with commissioned surveys

Schools and wider stakeholders expressed several benefits of working with universities to conduct health and well-being surveys including expertise, reputation, and quality.

*“Knowledge that they [schools] will be getting really topical information, learning from quite renowned, maybe, experts in the field. It’s obviously accredited with an education establishment, such as the university. Just that robustness of it, that it’s evidence-based, but also, in terms of academia, it’s also recognised by the bodies that schools would know about. I think that would be helpful, just in terms of gauging support.” (LA 1)*.

Despite these benefits, several local authorities (LAs) and schools across the South West have existing relationships with commissioned survey providers which presented as a very clear barrier to involvement in SW-SHRN. Strengths of commissioned surveys included supplying raw data to schools, having run the same survey for several years and therefore allowing year-on-year comparison, as well as commissioned surveys being open to all year groups and running in both primary and secondary schools. Because of these established relationships with commissioned survey providers, stakeholders described SW-SHRN needing to go above and beyond what these existing surveys were providing.

*“You’ll kind of come up against the other suppliers of school surveys in the region, no doubt…I suppose if they’re funding them at the minute, you would have to pitch them the advantage of moving from a supplier that they have been used to using.” (KS 6)*.

*“You’d be coming as the new person versus the people who they currently have a relationship with, who they are already working with and they probably are satisfied to some extent…cost saving would be a good argument.” (LA 3)*.

LAs also described the importance of having input to survey content and a level of ownership over the data which many had with their commissioned survey providers. To compete with other survey providers, it would be important for SW-SHRN to offer local authorities the ability to shape the content of the survey and for them to be able to interact with and analyse the data for their own purposes.

*“Local authorities really like having control over things… I think to kind of lose that control and not have that…I don’t know if ownership is the right word, but not have that flexibility, not have that entire control over that process or the content I think is something that might be a bit difficult for a local authority.” (LA 3)*.

*“We always make some recommendations about local questions, that we’d like to see…It’s always a negotiation, and we never get all the questions that we want, but usually, we get some of it…So, the possibility of adding a small number of local questions would be good.” (LA 5)*.

#### Scarcity of school time and resource

A key issue for schools was a lack of time and resource outside of the planned curriculum. Staff mentioned difficulties organising the survey around planned lessons for two entire year groups and the need for a dedicated member of staff, as well as administrative or IT support, to assist with this.

*“So logistically organising something, I mean in a school it’s always difficult…but just logistically pulling two whole year groups out of lessons to do the survey it takes time. You’ve got to organise that. And I think having a member of staff that takes responsibility for it is the only way that that’s actually going to happen.” (SC 13)*.

The opt-out consent process that was managed by the research team benefited schools and reduced burden.

*“I must say though the way that you handled the opt out thing was a great help to this school. I think if we were having to deal with consent, it would have been a nightmare….Actually, en masse, most parents are happy for their children to take part in something like that. If you’d said to me you need to make sure that you get parental consent and student consent for all 280 year 8s, and 260 year 10s, then I would have been pulling my hair out.”(SC 9)*.

Another benefit was schools having access to University iPads to support data collection and to ease the pressure on booking computer rooms for the survey.

*“I think having the iPads as well was a massive, massive bonus because we’re such a big school, and the amount of classes that use the computer spaces it was just going to be nearly impossible to get a day where we’d get most of the year groups done.” (SC 9)*.

### Theme 2: key facilitators to SW-SHRN: providing evidence-based support for schools

A key feature that attracted schools to join SW-SHRN was access to evidence-based support for schools. This theme was broken down into 4 subthemes: (1) Improved knowledge of children and young people’s health and well-being; (2) Feedback reports and benchmarking; (3) Data to inform interventions & monitor impact and (4) Interpretation and implementation.

#### Improved knowledge to facilitate change

Stakeholders referred to the importance of measuring young people’s health and well-being at scale to improve their overall knowledge and understanding of this population in order to create meaningful and targeted change in areas of need.

“*For us it [survey] has the potential to be really helpful, because we just do not have any other way of soliciting the views of such a wide group of the population, because we have not done a questionnaire, like so many other areas. So to be able to get such a large amount of data is very helpful.” (LA 2)*.

*“There is massive potential for school improvement and the use of the network to grow- measurement practices to grow data use, evidence-informed decision-making practices. Which, in turn, should improve children’s outcomes. There is no reason why it wouldn’t, if you are understanding needs well and finding evidence-based ways to respond to those needs.” (KS 2)*.

School staff also discussed how the data could identify groups at need and indicate health topics that may require more focus, as well as hopes that the data may facilitate more open conversations about health with students.

*“This survey has helped identify particular groups of students and areas which we could now work on rather than just shooting in the dark at what we could do and offer the students.” (SC 9)*.

*“I hope it will open up more conversations with our students, and us knowing what areas they need support on, and then being able to target these areas without saying to them, “What do you need support with?” Instead, actually saying to them, “We’re going to do this topic,” and knowing that this topic has been highlighted through the study that that has affected them.” (SC 15)*.

#### Feedback reports and benchmarking

Another key benefit of SW-SHRN to schools was the use of individualised school feedback reports and infographics as well as the use of benchmarking data to allow schools to compare their results with other participating schools across the South West. One teacher also reflected on the data challenging their assumptions.

“*I really liked the graphic, that was probably the most powerful part of it. The breakdown of the questions, that was really fascinating, in having the bar graph for the pupil premium versus the non-pupil premium and free school meals, boy and girl ratio, that was really, really powerful. There were some things where we probably assumed things about, say, a group of boys in year ten and actually it’s come back the opposite of what we assumed.”* (SC 9).

*“I thought the report was brilliant, it was really clear. I really liked the benchmarking that you did and the breakdown of boys and girls in different groups. That was really useful. That’s not something we’ve had from the [commissioned survey provider] before. So I was able to identify Year 10 boys that are pupil premium students, we’ve got a real problem with this. Being able to identify that specifically is really, really useful.” (SC 13)*.

Although benchmarking was considered a benefit by all schools, local authorities highlighted possible sensitivities when schools fall below average when benchmarked on certain health areas.

*“I thought it was really cool to think about doing really big comparisons across much larger sets of data. But then again…schools are really sensitive to that. So, schools are really happy when they’re doing better than other places, but obviously they’re not so happy when they find out they’re not doing that as well.” (LA 3)*.

#### Data to inform interventions

Using SW-SHRN data to inform potential interventions was vital to schools and local authorities. Academy staff also discussed the usefulness of having data to inform intervention suggestions to their academy trust and senior leadership team.

*“We want to know where the kids are at, where their needs lie, and what needs we are meeting, and then what needs we need to work on. In getting interventions to go through our trust, and to go through our SLT (senior leadership team), if there’s data provided to say, “We need to do this intervention, because this data has shown us that that is what we need to work on,” it’s so much more meaningful to be able to approach an intervention with the senior members of staff”* (SC 15).

*“I think it’s using [the data] as we move forward, it’s those conversations about how do we benefit the well-being of the students…what changes can we make, or what sorts of things do we need to look at bringing in?” (SC10)*.

One local authority also discussed involvement of the University to either develop an intervention or suggest existing interventions.

*“I do wonder whether the university can see that there are common issues, which are cropping up across several areas. And whether or not it would not be possible for the university to either develop an intervention itself, which is then bought in by different areas, or whether the university…could suggest interventions which already exist.” (LA 2)*.

### Theme 3: effective dissemination of findings

Several suggestions were provided in terms of how to effectively share network findings to make them accessible and meaningful to schools, students, parents/caregivers, and wider stakeholders. This consisted of supporting schools to make use of their individual data reports as well as how best to share findings more broadly across the network and with a wider community of stakeholders. This theme was divided into four subthemes: 3.1 Interpretation and implementation; 3.2Embedding the findings with existing evidence and policy; 3.3) Centralised online platform and direct personalised communication; and 3.4 Sharing findings with young people and families.

#### Interpretation and implementation

The need to support schools with interpreting network findings as well as supporting with recommendations for interventions to implement. This included helping to signpost schools to evidence-based interventions, resources and organisations, as well as supporting the school to better focus the curriculum to cover focus areas addressed in the feedback reports.

“*It would just be thinking about what support they [schools] have afterwards. Because it is often quite hard to interpret that kind of data, if that’s not what your job is. And then to decide what the next steps are and to drive real change. I think there is that support training…that support to translate data. So, improving school level skills in using the data and interpreting the data. And potentially that signposting and some sort of gateway through to the ‘what works’ evidence as well.”* (KS 1).

Teachers also suggested researcher-provided tailored resources for each health area covered in the survey to allow schools to directly act on the findings.

*“I mean really if I was going to attain my dream it would be to have linked to the report if your school is below average in this, here are some resources to address it for all of the different areas that you survey on.” (SC 13)*.

#### Embedding the findings with existing evidence and policy

Stakeholders discussed the importance of integrating network findings with the existing evidence-base and existing policy and practice. Therefore, rather than just being sent SW-SHRN data alone, schools and stakeholders were keen for researchers to put the data into context for them as well as circulating suggested resources. Schools did not want a one-off interaction, but were keen to keep up to date with the latest evidence and policy, highlighting the importance of the network being ‘live’ and regularly updated.

*“Like any network…providing a kind of noticeboard, really, about what the latest developments are in policy and strategy; the findings about what the evidence base is. If the network is a resourced one where it can host, and it becomes trusted and it has got a website, webpages or a newsletter, and we can post things in there about developing evidence, research.” (KS 4)*.

Schools suggested that the network could act as a signposting service between national policy, emerging initiatives and schools as well as helping schools understand current local statistics to provide local context to their report data, as well as providing suggested resources.

*“Within mental health, and that broadness of the PSHE and the network, there are a lot of changes coming out nationally, and things that are being pushed on in terms of awareness, so the opportunity to regularly engage with those, so having article postings.” (SC 1)*.

*“I suppose resources; if you could have some current statistics for the area, so on percentages, perhaps, even from a social norms perspective, so: “Actually, not as many students or kids as you think are drinking or smoking.” You know? And has mental health in the South West declined? Has it increased? What resources are out there?” (SC 7)*.

#### Centralised online platform and direct personalised communication

Stakeholders offered a range of methods and platforms for sharing data, the primary preference was having an interactive online hub to store updates, network data, ongoing network events, as well as providing an overview of the wider evidence and policy context as discussed above.

*“I think having it as a bit of a hub, almost… And then I think just having ‘planned events’…to say ‘this is what we have got planned moving forwards’…I don’t know, data protection-wise, but having somewhere to go on and look at maybe comparing ourselves against other schools. Just having a normal website and then, for the schools that are part of the network, having an access-only part as well. Just combining the two.” (SC 10)*.

Schools were also keen to receive evidence summaries, headline findings, and regular updates about the network through email bulletins or newsletters.

*“Being in a school you can become very insular and focus on your own things, but actually having a quick bulletin of ten things that have happened to help with well-being in other schools just gives you that quick, ‘Right, actually, that’s a good idea, we could try that maybe in our school’. I think anything like a bulletin that’s clear…that’s got headline facts or figures, or good practice…that’s probably the most valuable thing that we could receive.” (SC 9)*.

*“I know that our school does and I know a lot of schools have these rolling screens, so television screens that can have rolling data…simple things like, “Did you know less than – I don’t know – 5% have tried smoking or more than 95% of students in the South West have never done this?“ One headline at a time… that gives students a chance to actually take in the information.” (SC 7)*.

Stakeholders also suggested the use of policy briefings and success stories or case studies of schools implementing change as a result of SW-SHRN findings.

#### Sharing findings with young people and families

Stakeholders suggested a number of people to involve when disseminating findings, but particularly emphasised the importance of sharing findings with young people and using a ‘you said, we did’ approach to allow young people to see the results of their survey responses in action.

*“Children and young people get asked to do loads of surveys… there needs to be a version of “you said” and “what we’re going to do or what we can do to help support what you have said”. The school gets theirs [data], but then sometimes we think about that third part of the triangle, which is the young people who have done it.“ (LA 4)*.

As well as sharing the findings with young people, stakeholders also highlighted the importance of sharing findings with parents and school governors in an accessible way as well as using the findings to shape school policy, curriculum and development plans.

*“Put stuff in the parent newsletter over the next year or two. Just drip feed a few facts about what times children go to bed, or what they’re eating, or if they’ve had breakfast. Things that parents might be interested in.“ And obviously, the good news, share it with governors. Try and get something in the school development plan, share it with the PSHE coordinator. Just get it out there.” (LA 5)*.

Although one local authority staff member suggested sharing ‘good news’ with governors, careful consideration is needed on how to meaningfully share the challenges schools may be facing, for example when they are below the benchmark in a particular health area.

### Theme 4: longer-term facilitators: ensuring sustainability

Participants also discussed a number of longer-term facilitators which focussed on the sustainability of the network and potential ideas to expand the grown of the network in the future. This comprised of four subthemes: (1) Keeping schools engaged; (2) Repeat surveys and evaluating intervention impact; (3) Informing school inspection frameworks and (4) Expanding reach and enhancing accessibility.

#### Keeping schools engaged

Schools and stakeholders highlighted the importance of maintaining contact over time and continuing to provide updates and share latest findings to keep people engaged during the gap between biennial surveys. This links to suggestions for an online hub for schools to keep up to date with network activity and latest evidence. Schools also mentioned the issue of staff turnover and the need for regular communication to maintain links. Schools and stakeholders also noted the importance of ongoing involvement of young people in shaping the network and its resources.

*“It’s keeping them in the loop regularly, just to remind them about this network…, it’s difficult, isn’t it, because it’s like a balance between keeping them in the loop and keeping them up-to-date…but also not asking them to do anything because you don’t want to burden them.” (KS 5)*.

*“I think just having an ongoing conversation, and issuing reports, and picking out findings from the previous survey keeps it alive. It reminds people of what the survey was, the value of it, what it can do, how you can respond to it.“ (LA 5)*.

It was also acknowledged that schools would like to be recognised for their involvement in the network which was another means of keeping schools engaged long-term, for example if they were working towards an accreditation.

*“I suspect they would quite like to be named because they’ll be seen as kind of trailblazers for working on this, and with the push, like we say, about the curriculum changes and that kind of thing, being shown as one of the front-runners of linking into this kind of network could well be quite a kind of status thing for the schools.” (KS10)*.

#### Repeat surveys and evaluating intervention impact

Something that school staff and local authorities noted as lacking is the ability to evaluate the impact of health and well-being interventions. Stakeholders therefore discussed the value of monitoring change over time and supported the suggestion of repeat biennial surveys to monitor change when new policies or interventions are implemented. One school detailed how they were always looking to ‘*monitor, track, improve and reflect*’ (SC15) as a type of audit and feedback approach. However, repeat surveys would need to be carefully planned given the existing barriers discussed regarding lack of time and burden on schools.

*“How can you use the data to inform a strategic approach to health and well-being and monitor it. I guess that’s the thing, monitor your changes. So, you change something, have you had the effect you wanted to have? This is where they were at, this is what they implemented, this is the benefit.” (KS2)*.

*“I think the value of the questionnaire that is being done, the value very much lies in repeating it, doesn’t it? Because it is not much use to schools if they do loads of work to address an issue it is not particularly helpful if they cannot find out whether they have made a difference or not.” (LA2)*.

#### Informing school inspection frameworks

Stakeholders provided their thoughts on health and well-being becoming a bigger focus in future Ofsted (The Office for Standards in Education; the government department responsible for inspecting education institutions in England) frameworks and how this may impact sustainability of the network. Overall stakeholders agreed that if it were to become a more central focus this would lead to increased buy-in to the network. Stakeholders also mentioned the potential for Ofsted to use SW-SHRN data to target particular areas for inspection. However, some stakeholders were hesitant on schools being scrutinised on health and well-being outcomes by Ofsted due a wide range of factors external to the school impacting on this. Overall, stakeholders saw promise in schools being able to demonstrate awareness of need and targeted action to Ofsted inspectors as a result of SW-SHRN data.

*“The survey would help identify those needs [child health needs], and you could say that the schools’ PSHE programme is informed by the data they’ve collected about their needs and behaviour. It would be really good to be able to present that to Ofsted, saying we’re aware of the needs of our children, and we’ve responded as a school to the data which we’ve collected, just like they would for data about academic subjects or anything else.” (LA 5)*.

*“I think it could go both ways, couldn’t it? It depends quite how they mandate it, whether they mandate that it must be a particular measure or a particular time…But on the other hand, if schools have to evidence that they are already doing something, actually that might really help buy in to this. I hope Ofsted would not go down a line of saying, “It has to be this measure at this time,” because I do not think there is a perfect measure out there”. (KS 7)*

#### Expanding reach and enhancing accessibility

In terms of the network being sustainable long-term, a common query or suggestion amongst stakeholders was whether we could extend the survey to include primary schools, as well as offering schools the option to run the survey with all secondary school year groups. The value of reaching older children (16 +), as well as children who are home schooled or attending alternative provision academies was also mentioned. Surveying children as early as possible e.g., in nursery and/or primary was seen as optimal as the data would support early preventative work.

“*There are many thousands more primary schools than there are secondary, so you’re able to reach a much larger audience. But most importantly, it is preventative work. So, I would be looking at nurseries as well. If you’re not surveying kids until they’re in Year 8, that is quite late. So, I would be interested in trying to understand more, as early as possible. It will presumably give you much more data and greater benchmarking and ultimately more power.” (KS 1)*.

*“I think in terms of provision for children who are…home educated, just being aware that we have a virtual school and the…Alternative Provision Academies, for children who have been expelled or excluded. I think, for us, it’s just making sure that we tailor any resources and needs to those more niche audiences. I think schools are always looking at how they are as inclusive as possible.” (LA 1)*.

As well as future expansion, schools discussed the importance of the survey being inclusive and accessible for students with special educational needs or lower reading abilities in mainstream secondary schools.

*“Having the option to have it read to them probably would help…I know it is hard if you’re using validated surveys, some of the language is a little bit inaccessible. So I think it’s got to be inclusive and accessible.” (SC 13)*.

## Discussion

The aim of this study was to identify the key barriers and facilitators involved in setting up a regional SHRN in the South West of England and to identify opportunities for refinement of the network to enhance its sustainability. We identified four key themes (1) Key barriers to SW-SHRN; (2) Key facilitators to SW-SHRN: providing evidence-based support to schools; (3) Effective dissemination of findings; and (4) Longer-term facilitators: ensuring sustainability.

Barriers incorporated pressures on school time, different levels of prioritisation on student health and well-being in comparison to academic attainment, and competing with existing commissioned health and well-being surveys. These barriers are consistent with a recent systematic review of sustaining school-based mental health and well-being interventions [21]. The review found that competing priorities and responsibilities often led to intervention delivery challenges and also highlighted the need for school interventions to be easy to use or implement and well-organised. These two findings are in line with our study results relating to competing school priorities and discussion of time pressures and reducing burden on schools.

Although the links between health improvement and educational attainment are well-evidenced within the academic literature, it seems particularly important to clearly communicate this link to school staff, local authorities and academy trusts, particularly with reference to our findings regarding competing priorities between health and well-being and academic achievement and reassuring schools that focussing on health and well-being is not diverting resource away from the core curriculum and attainment. Previous research from the Welsh SHRN demonstrates emerging evidence of better educational outcomes in schools with more extensive health improvement policies and practices [22] which is another important factor when communicating the benefits to schools of participating in a SHRN.

To address these barriers, SW-SHRN aims to provide collaborative opportunities for schools to share best practice between one another and across different local authorities in an effort to create an active learning network. By building an active learning network that multiple partners benefit from (similar to the Welsh and Scottish model) we hope to make the research/survey burden worthwhile for schools and go above and beyond existing survey provider offerings. As SW-SHRN grows and more schools participate, we hope the network can offer a more standardised approach to health and well-being surveys across the region and in turn reduce the number of survey requests that secondary schools receive. There are also unknowns on how commercial survey companies deal with ethical requirements, data security and ownership of data, therefore a university-led SHRN hopes to provide schools a robust and secure method of collecting student data.

It will be important to take an inclusive approach in terms of promoting the network and recruiting to the network to ensure all the relevant education infrastructures are incorporated to maximise the growth of the network given the diverse school system in England. Previous literature has evidenced that collaboration with the education sector is critical when developing health-promoting schools programmes [23].

A key facilitator to SW-SHRN is the ability to provide schools with evidence-based information to enhance their understanding of mental health and well-being in school populations as well as identifying health needs and challenges, for example subgroups of students requiring more support or intervention. What seemed to set SW-SHRN apart from existing school surveys was the individualised feedback reports. Within these reports, schools valued the use of benchmarking data to allow them to see where they sit in the context of all participating schools in the region as well as the break down of data by gender, year group and socio-economic status. In turn, these detailed reports aim to allow schools to more effectively target health areas both within the curriculum as well as through targeted resources and interventions.

There was also a need to make network outcomes and impacts clear to schools, local authorities, and wider stakeholders, which echoes findings from intervention developer perspectives of evidence-based interventions in schools [24]. A common suggestion to make outcomes and impacts visible was to provide an online platform containing network data which incorporates evidence summaries and policy impacts which would be available to schools and all key stakeholders. A key finding was the need to embed SW-SHRN findings in the wider evidence base and put the findings in context of existing knowledge of young people’s health and well-being, as well as linking findings to existing policies and practice [24]. This also aligned with supporting schools to interpret their data and implement meaningful change, schools felt they required support from the research team to translate survey data into action.

Schools and stakeholders reflected on how to ensure sustainability of SW-SHRN. Sustainability within the context of the network refers to how to sustain the growth of the network (number of schools, academy trusts and local authorities involved), sustaining active involvement from participating schools (e.g. engaging in repeat surveys and acting on findings) and sustaining meaningful collaborations between stakeholders. Stakeholders discussed the importance of the network maintaining a wider systems perspective, continued conversations with key stakeholders and embedding network findings within wider national policy.

One important aspect relating to sustainability is the role future Ofsted frameworks could play in sustaining SW-SHRN if health and well-being were to form a larger part of future frameworks. Stakeholders saw value in making use of SW-SHRN data to inform student need and modifying provision accordingly (e.g. PSHE curriculum), which could then be presented to Ofsted to showcase meaningful health and well-being activity. An important area of future research could focus on how best to mandate routine monitoring of health and well-being provision in schools.

Findings revealed the potential SW-SHRN data holds to support and inform both regional and national policy and planning, and the implications this may have on who may support funding the network in the future. Working at a systems level has been effective for the Welsh School Health Research Network, their network has been effectively embedded into the system and plays a key role in national and regional planning [14].

The suggestions from participants regarding joined up working, influencing questions to drive policy, and understanding challenging areas through comparison with other schools demonstrate the need for connecting multiple systems and structures and a requirement for the network to monitor and intervene at multiple levels (e.g. school level, local authority level, government level). Together, these suggestions reflect the need for the network to take a systems-based approach.

A possible area of future expansion for the network noted by several stakeholders could be the inclusion of primary schools, as well as 16+, and alternative provision settings, to allow SW-SHRN to provide a more complete picture of health and well-being across all school settings and in all age groups. Primary schools were of particular interest as an area for expansion, both to allow for earlier intervention, to allow for longitudinal tracking of health and attainment outcomes and also due to many multi-academy trusts comprising of both primary and secondary schools and therefore wanting a network that was accessible for all of their schools. However, expanding to primary schools would need careful consideration, particularly in terms of how to sustain such a large network if expanded given the barriers identified so far.

Sustainability of public health interventions in schools remains relatively underexplored in comparison to health care and a recent review highlights particular difficulties with retaining senior leadership contacts given frequent staff turnover in schools [25]. Additionally, it will be important to refine definitions of sustainability relating to SW-SHRN as the network continues to develop [26].

One incentive to join the network could lie in its multiple forms of research participation, particularly for those schools who are less active in research. SW-SHRN offers involvement in a population health survey, 1:1 feedback on a tailored school report, qualitative interviews and focus groups with young people, as well as the school environment survey that may help schools reflect on their current health and well-being policies. A possibility for the network as it develops could be offering schools flexibility on which aspects of research they participate in.

An important overall finding from this study was the general unified opinions or advice given from key stakeholders, suggesting agreement and consensus around the importance of routine collection of health and well-being outcomes in young people. However, there were varied opinions and priorities across individual schools, particularly how schools would make use of SW-SHRN data and how much support schools felt they needed from the University in making meaningful changes as a result of the data they received from the network. This reinforces the individual nature and unique set-up of each school or academy and the need to offer a flexible and tailored research agenda to meet individual school needs. SW-SHRN in the future, for example, could offer different levels of school involvement depending on individual school preferences.

Findings from this evaluation will be used to develop, adapt and enhance the expansion of School Health Research Networks in England, with particular focus towards creating meaningful change in schools and supporting schools to effectively make use of the data generated from these networks. SW-SHRN will continue to routinely seek feedback from participating schools, local authorities and academy trusts to continue refining the model and prioritise areas of future expansion.

### Strengths and limitations

This is the first regional School Health Research Network to be set-up in England. This study benefits from seeking perspectives from a wide variety of school staff, six different local authorities across the South West, as well as advice from a wide range of relevant stakeholders including government departments, charities, researchers, and existing providers of health and well-being initiatives for young people. However, some limitations must also be acknowledged. Although this pilot study tests a regional School Health Research Network in the South West of England, school staff and local authority interviews only covered seven of the 15 local authorities in the region, therefore the findings may not translate to the whole region and it will be important for future SW-SHRN recruitment to target these remaining eight local authorities to gain their perspectives. Another limitation is that only one individual per school and local authority were interviewed which means we were not able to explore how far there were diverse opinions within schools or local authorities. Future work could benefit from the use of focus groups to allow discussion between members of staff and perhaps include combinations of school staff, local authority staff, and wider key stakeholders to encourage conversation around differing viewpoints.

## Conclusion

To ensure effective implementation and sustained growth, School Health Research Networks in England need to provide clear benefits to schools and ensure participation is not overly burdensome. Schools should be provided with detailed data reports to improve knowledge, facilitate change and inform interventions, and should be supported in interpreting report findings in order to take meaningful data-driven action. The network should develop in partnership and close communication with existing organisations and service providers to maximise relevance, avoid repetition and become meaningfully embedded in existing policy and practice.

## Electronic supplementary material

Below is the link to the electronic supplementary material.

Supplementary Material 1

## Data Availability

The datasets used and analysed during the current study are available from the University of Bristol data archive, https://data.bris.ac.uk/data/.
