# Supplementary material for: Implementing a regional School Health Research Network in England to improve adolescent health and well-being, a qualitative process evaluation
Source: BMC Public Health. 2023 Apr 23;23:745. doi: 10.1186/s12889-023-15713-9 (PMC10122722; doi:10.1186/s12889-023-15713-9)
Supplement: Supplementary file 1 — Supplementary Material 1 [file 12889_2023_15713_MOESM1_ESM.pdf]

Additional File 1: SW-SHRN School Contact Interview Topic Guide

Background

1. What is your role within the school?
    a. How did you (personally) come to be involved in the Network?
    b. Did you have authority over whether your school participated in the Network or did you have to consult with other staff/SLT members?
2. Could you briefly describe your understanding of the Network?

3. What was it that interested you and/or your school in participating in the Network?
    a. How did being part of the Network align with your school's priorities and culture?
    b. Your school was offered a small gratitude payment of [£200 update] for taking part, how did you feel about this - was this an incentive to take part?

Logistics

1. What did you think about the way your school was recruited to the Network?
   PROMPT: We contacted the school via email and sent an information brochure containing details of the study and Network.
    a. How helpful was the information you received about the study?
       PROMPT: Specifically the School Information Brochure.
    b. How can we improve the recruitment process, what would be helpful for recruiting schools?
        i. Are there barriers to taking part that we should be considering?
        ii. When is the best and worst time of year to be approaching schools about joining the Network?

2. What are your thoughts on the Network website?
    a. If they have not accessed the website: What information would you like there to be on the website?

3. A student can be opted-out of the study by themselves or by a parent/guardian. If the student is opted-out of the study by a parent, but the student wishes to take part, what do you think should be the course of action?

4. We collected student data in your school [delete options as appropriate]:
    - In school/class with an external researcher present to lead data collection (using either school computers, researcher tablet devices or a combination of both)
      OR
    - In school/class with a teacher administering data collection
      OR
    - At home data collection during an online learning session and in class with vulnerable children

   What were your experiences of data collection with the students?
    a. Can you think of anything that may improve this process?

5. What would be your preferred method of student data collection post-pandemic?

If interviewee completed the staff questionnaire (school environment survey) themselves…

6. How did you find the questionnaire you completed for the study?
    a. What changes would you make to the questionnaire to improve it, if any?
        i. Did you feel any questions were missing that you would have liked to receive data on, if so, what were these?
    b. What were your thoughts on how you completed it – i.e., over the phone with a researcher?

Being part of the NETWORK

1. What benefits are you hoping to get out of being part of the Network?
   PROMPT: Obtaining data about your students, having support to make changes to benefit your students' health and wellbeing, newsletters, training courses or outreach days.
    a. What would you like to see from the Network, aside from the feedback report and meeting to discuss the findings of the school surveys?

If the interviewee has not yet received their student report…

2. You will soon be receiving your student health and well-being report with the results from the student questionnaires that the Year 8s and 10s completed as part of our research. What kind of information would you like to see in the report?
    a. How would you like this presented?

3. The second report you receive will benchmark your schools' data to average data from all participating schools, what are your thoughts on this?
    b. Is there any particular information or schools you wish to be benchmarked against?

4. What do you hope to gain from the 1:1 meeting that is planned with the senior researcher to discuss the findings?

5. What do you aspire to do with the feedback we provide you?
   PROMPT: Think about potential changes you could make to school policies and interventions
    c. Do you envisage needing any help or support to carry out any plans?

If the interviewee has received their student report…

2. You received your first student health and well-being report with the results of the student questionnaires that the Year 8s and 10s completed as part of our research. Can you tell me what you thought of the report?
   PROMPTS: Think about what and how the information was presented and the length of the report.
    a. Are there any discriminators (e.g. ethnicity or disability) that you would be interested in having shown in the report?
    b. How would you improve the report to ensure schools fully benefited from it?

3. The second report you receive will benchmark your schools' data to average data from all participating schools, what are your thoughts on this?
    a. Is there any particular information or schools you wish to be benchmarked against?

If they have not had their 1:1 feedback meeting:

4. What do you hope to gain from the 1:1 meeting that is planned with the senior researcher to discuss the findings?

5. Have you taken any action as a result of the feedback we have provided you?
   IF YES: What action have you taken?

   IF NO: What do you aspire to do with the feedback we provide you?
   PROMPT: Think about potential changes you could make to school policies and interventions
      a. Do you envisage needing any help or support to carry out any plans?

If they have had their 1:1 feedback meeting:

5. How did you find your 1:1 meeting with the senior researcher to discuss findings?
   a. What was beneficial about it?
   b. How would you improve the meeting?
   c. Was there anything that did not need to be covered?

6. Have you taken any action as a result of the feedback we have provided you?
   IF YES: What action have you taken?

   IF NO: What do you aspire to do with the feedback we provide you?
   PROMPT: Think about potential changes you could make to school policies and interventions
      a. Do you envisage needing any help or support to carry out any plans?

7. What plans, if any, do you have to circulate or discuss the findings with any of your internal stakeholders, e.g., parents, students or governors?

8. Is there anything we could do or any materials we could provide you to aid your ability to integrate the Network into your school?

Sustainability

1. Would you be happy to participate in the next round of surveys within the Network?
   a. If not, can you think of why?
   b. What could encourage your continued participation in the Network?

2. How, if at all, do you think that academy status/affiliation could impact on whether schools take part in the network?

3. How, if at all, do you think whether a school's local authority supports and participates in the network could impact on whether schools take part in the network?

CLOSING

- Is there anything else that you think is important that we should know about regarding what we have discussed today?
- Do you have any questions for me?

Additional File 2: SW-SHRN Key Stakeholder Interview Topic Guide

Introduction for Local Authority contacts already participating in the project

Thank you for taking part in the interview today. As you will know, your local authority is collaborating with University of Bristol to create a new School Health Research Network with secondary schools in the South West of England. Some secondary schools in your area have taken part already. We are interested in your opinions on the Network and would like to discuss these with you.

This interview will broadly consider the following themes:

1) *Your thoughts and opinions on the Network*
2) *How you can benefit from the Network*
3) *How you could potentially support the Network long-term*

Introduction for non-LA stakeholders with prior knowledge of the project

Thank you for taking part in the interview today. You have been involved in research being carried out by the University of Bristol to create a new School Health Research Network with secondary schools in the South West of England. We are interested in your opinions on the Network and would like to discuss these with you.

This interview will broadly consider the following themes:

1) *Your thoughts and opinions on the Network*
2) *How you can benefit from the Network*
3) *How you could potentially support the Network long term*

Introduction for non-LA stakeholders with no prior knowledge of the project

Thank you for taking part in the interview today. I would like to explain a little bit about the School Health Research Network before we begin and have created a couple of slides to help me do this.

[Share slide set with participants]*:*

We have recently undertaken a pilot study involving the creation of a new School Health Research Network with secondary schools in the South West of England. For this we asked secondary school children to complete a survey about their health and well-being, and a member of school staff to report on health promotion policies and interventions delivered in their school. The aim is to revisit schools every two years and create a longitudinal dataset on schools in the South West. Each school receives a tailored report on the health and well-being of their students, and a summary report of health policies and interventions being delivered across schools participating in the Network. Each participating local authority also receives a summary report on the data collected from schools participating in their area. The primary purpose of the Network is to help improve health and wellbeing and educational attainment in schools in the network by creating a collaborative framework to facilitate a long-term relationship between University research and key stakeholders in public health delivery for school-aged children. I hope this provides some context on the Network for you. Do you have any questions?

This interview will broadly consider the following themes:

1) *Your thoughts and opinions on the Network*
2) *How you can benefit from the Network*
3) *How you could potentially support the Network*

Background on SW-SHRN

Explain the Network further if needed and answer any participant questions.

Stakeholder context

1. Could you briefly describe your role and how it connects with school health and wellbeing?
    a. Do you have any direct influence over school health policies, interventions or commissioning? Please expand.

About the SHRN

1. Can you tell me your thoughts on the Network as a framework for improving school children's health?
    a. What potential do you think the Network has?
    b. Any limitations / challenges to consider?

2. How do you think you and your organisation could benefit from the Network?
   PROMPT: That could be partnership working, use of the data, activities beyond the survey…

3. How do you think the Network could be utilised to its full potential for schools and those working in the context of school public health?
    a. What services could the Network provide?

Supporting the Network

1. What would encourage you to buy into and support the Network?
    a. How about supporting the Network on a long-term basis?

2. How do you think you could support the Network in terms of:
    a. Recruitment of and access to schools
        i. How do you currently influence schools?

3. How do you think you could support the Network in terms of:
    a. Funding the Network
        i. Would you have capacity to financially support the Network?
        ii. *If YES* – Under what conditions could you provide financial support?

Note to interviewer:<br>If the participant talks about or mentions collecting data from primary schools or early years (in response to this or any other questions), ask what the reasons behind this are.

        iii. Could this be long-term support or a one-off contribution?

4. How do you think you could support the Network in terms of:
    a. Dissemination of findings to schools and other stakeholders (i.e., how can you facilitate the sharing of findings to ensure the outcomes reach a wide range of audiences)

Network output

1. What type of outputs would you like to see from the Network?
   PROMPT: Blogs, newspaper articles, policy briefings, evidence summaries

2. How do you think the Network could evolve to deliver other resources to support wider children and young people's health?

   a. How do you think stakeholders could use the findings to develop and implement health policies in school?

3. Health has recently become a compulsory part of the school curriculum. Expectations on the delivery of this curriculum may vary depending on school circumstance but should have begun teaching by the summer term of 2021.
   a. What are your opinions on health forming part of the inspection framework in England?
   b. How do you think this could influence schools signing up to the Network?
      i. How do you think the Network could support schools in relation to the new curriculum?

For Local Authority participants who have not received their report…

4. You will receive a report on the data from the schools in your area, specifically what kind of information would you like to see in the report?
   a. How would you like this presented?
5. The report, where appropriate, will also benchmark to other local authority data, what are your thoughts on this?
   a. How useful would this be to you?
   b. Is there any particular information you would like to be benchmarked against?

For Local Authority participants who have received their report…

5. You received a report on the data from the schools in your area, can you tell me what you thought of the report?
   PROMPTS: Think about -
   What and how the information was presented
   The length of the report
   The benchmarking data
   a. How would you improve the report to ensure that local authorities could fully benefit from it?

COVID-19

1. How do you think the Network may have helped support schools throughout the COVID-19 pandemic, including their COVID-19 recovery response?

Sustainability and scalability

1. How do you think we could encourage schools to sign-up to the Network?
   a. How could we ensure schools remain part of the Network long term?

2. Because of the COVID-19 pandemic, where possible we gave schools the option of having a researcher present in class to manage the data collection process. How valuable do you think it is to have researchers lead data collection within a school and why?

Closing

- Is there anything else that you think is important that we should know about regarding what we have discussed today?
- Do you have any questions for me?
